# Estimation Strategy Utilization Is Modulated by Implicit Emotion Regulation: Evidence from Behavioral and Event-Related Potentials Studies

**DOI:** 10.3390/brainsci13010077

**Published:** 2022-12-30

**Authors:** Chuanlin Zhu, Xinyi Zhao, Feng Lu, Yun Wang, Yuan Zhao, Dongquan Kou, Dianzhi Liu, Wenbo Luo

**Affiliations:** 1 School of Educational Science, Yangzhou University, Yangzhou 225002, China; 2 College of Educational Science, Taizhou University, Taizhou 225300, China; 3 School of Foreign Languages, Suzhou University of Science and Technology, Suzhou 215009, China; 4 Police Officer Academy, Shandong University of Political Science and Law, Jinan 250014, China; 5 School of Education, Soochow University, Suzhou 215123, China; 6 Research Center of Brain and Cognitive Neuroscience, Liaoning Normal University, Dalian 116029, China; 7 Key Laboratory of Brain and Cognitive Neuroscience, Liaoning Province, Dalian 116029, China

**Keywords:** implicit emotion regulation, ERP, LPC (late positive component), estimation

## Abstract

A large number of studies have studied the influence of emotional experience on an individual’s estimation performance, but the influence of implicit emotion regulation is still unknown. Participants were asked to complete the following tasks in order: idiom matching task, multiplication computational estimation task (MCE task), gender judgment task (GJ task), and emotional experience intensity assessment task. The words matching task was adopted to achieve the purpose of implicit emotion regulation (implicit reappraisal and implicit suppression). Behavioral results showed that implicit reappraisal and implicit suppression equally contributed to improving an individual’s estimation speed (but not ACC (accuracy)). The MCE task related ERP (event-related potential) results showed that the influence of implicit emotion regulation on estimation consisted of two phases. In the first phase (encoding phase), implicit reappraisal both enhanced (larger P1 amplitudes) and weakened (smaller N170 amplitudes) an individual’s encoding sensitivity, while implicit suppression enhanced an individual’s encoding sensitivity (larger P1 amplitudes). In the second phase (estimation strategies retrieval phase), implicit reappraisal (but not implicit suppression) cost more attention resources (larger LPC2 and LPC3 amplitudes). The present study suggested that both implicit reappraisal and implicit suppression contributed to improving an individual’s estimation performance, and the regulation effect of implicit suppression (vs. implicit reappraisal) was better.

## 1. Introduction

Previous studies showed that an individual’s estimation performances might be affected by emotion experience [1,2,3], age [4,5], cognitive load [6,7], cognitive style [8], and cultural differences [9,10], and so on. For example, a study [11] showed that when participants were asked to complete an estimation task with a specified strategy, their performances were influenced by their math anxiety level. To be more specific, compared with the high math anxiety group, the low math anxiety group showed higher ACC and shorter RT (response time); in other words, participants with low math anxiety performed better. Additionally, a recent study explored the influence of emotional experience under laboratory conditions on individuals’ performance of completing an estimation task [2].

Combined with the emotion priming paradigm, Liu et al. [2] asked participants to complete the MCE task and the emotion labeling task in each trial, under angry, fear, happy, and neutral priming conditions (the prime stimuli were facial expression images). In the MCE task, the multiplication estimation question and two alternative answers were presented on the screen simultaneously; participants were asked to find out the correct answer by using the down-up strategy (DU strategy, rounding the first operand down to the nearest decade, while rounding the second operand up to the nearest decade, e.g., doing 30 × 70 = 2100 for 32 × 67). In the emotion labeling task, participants were asked to judge which emotion was conveyed by the priming stimulus (facial expression image). The behavioral results showed that participants’ ACC of completing the MCE task under different emotion priming conditions showed no significant difference, while the corresponding RT under fear (vs. neutral) priming condition was longer, which indicated that fear priming reduced participants’ estimation speed. More importantly, this study indicated that not all negative emotional experiences (e.g., fear) were detrimental to participants’ estimation performance.

However, the result of Liu et al. [2] was based on explicit priming condition, so what influence does implicit emotional priming have on individuals’ estimation performance? To solve this issue, a newly published study [1] adopted the same experimental procedure and stimuli as Liu et al. [2], except they replaced the emotion labeling task with a gender judgment task, i.e., participants were asked to judge the gender of the facial expression image (priming stimulus). The results indicated that participants’ ACC of completing the MCE task was not influenced by the emotion type of the implicit priming stimuli, which was consistent with previous study [2]. However, the corresponding RT under implicit happy and fear (vs. neutral) priming conditions was shorter, which indicated that implicit happy and fear priming improved participants’ estimation speed. In a word, these studies [1,2] showed that an individual’s estimation performance was influenced not only by emotional experience, but also by emotional priming style (explicit vs. implicit). Can emotion regulation improve the adverse effects of negative emotional experience (for example fear) on individuals’ estimation performance? If so, what are the similarities and differences between the effects of explicit emotion regulation and implicit emotion regulation?

A recent study has explored the influence of emotion regulation on an individual’s estimation performance [12]. In this study, participants were required to complete the MCE task by using the DU strategy, under neutral, happy, and fear priming conditions, during which they were told to regulate their emotional experience by using a specified emotion regulation strategy (view, reappraisal, or suppression). Referring to previous studies [13,14], “reappraisal” refers to regarding oneself as a detached observer, and realizing that the emotion experience conveyed by the upcoming facial expression image has nothing to do with oneself; “suppression” refers to consciously suppressing one’s expression of emotional experience, elicited by the upcoming facial expression image, and trying their best to keep their facial expressions unchanged, thus leading others to be unable to recognize their emotional feeling through seeing their face. The behavioral results showed that participants’ ACCs of completing the MCE task under different emotion regulation conditions showed no significant difference. However, reappraisal (but not suppression) contributed to improving an individual’s estimation speed (shorter RT) under a happy priming condition, and both reappraisal and suppression contributed to improving an individual’s estimation speed under a neutral priming condition; however, an individual’s estimation speed under a fear priming condition was not influenced by emotion regulation. Additionally, the ERP results showed that the P1 amplitudes, elicited by using reappraisal (vs. view), were larger under happy and fear priming conditions, while the P1 amplitudes elicited by using suppression (vs. view) were larger only under a happy condition. Meanwhile, the corresponding N170 amplitudes were smaller when using reappraisal and suppression (vs. view) under a happy priming condition. Previous studies showed that the P1 component was an effective indicator of indexing attention to digit-pattern and their potential numeric meaning, and the amplitudes of P1 would be enlarged by attention; it may be an early indicator of brain activity when participants complete an estimation task [1,15,16], and N170 may be an effective indicator of indexing the digital encoding of the computing task [17,18]. Therefore, the P1 and N170 related results indicated that reappraisal and suppression contributed to encoding the estimation question. Additionally, the corresponding LPC amplitudes under the reappraisal (vs. suppression) condition were smaller, indexing less cognitive resource cost because previous studies showed that LPC seems to reflect the retrieval of procedural strategies, and is an important indicator of cognitive resource allocation, with easy (vs. difficult) computing problems which cost less cognitive resources [19,20]. Totally speaking, both reappraisal and suppression contributed to improving an individual’s arithmetic performance, and the regulation effect of reappraisal (vs. suppression) was better.

The dual-process framework theory proposed that both explicit and implicit emotion regulation were important for us, though researchers paid more attention to explicit emotion regulation in previous studies [21]. Explicit emotion regulation (also called controlled/conscious emotion regulation) refers to effortful attempts to change the time course and intensity of emotional experience and emotional responses [21]. Implicit emotion regulation refers to unconsciously goal-driven change to any aspect of an individual’s emotion experience and experience responses, while paying no attention to the process of regulating their emotions and without engaging in deliberate control. Therefore, implicit emotion regulation is also called automatic/unconscious emotion regulation [21,22]. Methods used to induce automatic emotion regulation include the sentence unscrambling task [23,24], word matching task [25,26,27], and implementation intention paradigm [28,29]. For example, adopting the word matching task, Yang et al. [25] investigated the influence of implicit emotion regulation on outcome evaluation. In this study [25], participants were required to complete the word matching task (two conditions: priming emotion regulation condition, and control condition) before completing the monetary decision-making task. In the word matching task, participants were required to select between two alternative words to match the meaning of the goal word. In the monetary decision-making task, participants were asked to select between two alternatives, which means that they would have won or lost different amounts of money, and then they were required to evaluate their emotional experience intensity in relation to the outcome. The results showed that priming emotion regulation contributed to reducing an individual’s emotional experience intensity when participants won money. Meanwhile, under the priming emotion regulation condition (vs. control condition), the feedback-related negativity amplitudes were reduced, which indicated that the word matching task modulated motivational salience of outcomes. Collectively, these studies mentioned above indicated that implicit emotion regulation can improve an individual’s emotional experience and cognitive performance.

To our knowledge, only a few studies have studied the relationship between emotion regulation and an individual’s arithmetic performance [30,31]; therefore, does implicit emotion regulation contribute to improving an individual’s performance of completing estimation tasks? In order to explore the relationship between implicit emotion regulation and an individual’s estimation performance and the potential neural mechanism, a 3 (implicit priming emotion type: fear, neutral, and happy) ×3 (implicit emotion regulation strategy type: view, reappraisal, and suppression) within-subject experimental design study was adopted. In the present study, participants were asked to complete the MCE task by using DU strategy (e.g., by doing 30 × 70 = 2100 for 34 × 67) under different implicit emotion priming conditions, during which they were asked to regulate their emotion by completing the idiom matching task. Please refer to the Methods section for details. Considering previous studies showed that participants’ ACC of completing the estimation task were not influenced by emotion priming [1,2], and the dual-process framework theory proposed that both explicit and implicit emotion regulation contributed to improving individuals’ cognitive performance [21], we hypothesized that participants’ RT (but not ACC) of completing the MCE task would be reduced by implicit emotion regulation (hypothesis 1). Additionally, considering a large number of previous studies showed that reappraisal contributed to improving an individual’s cognitive performance, along with less cognitive resource cost than suppression—i.e., the regulation effect of reappraisal was better than suppression [12,32,33]—we hypothesized that both implicit reappraisal and implicit suppression contributed to estimation question encoding (enlarged P1 and N170 amplitudes). Additionally, individuals who complete the MCE task under implicit reappraisal (vs. implicit suppression) condition cost less cognitive resources (reduced LPC amplitudes) (hypothesis 2).

## 2. Methods

### 2.1. Participants

Referring to a previous study [34], a total of twenty-six (14 women) students were randomly recruited by posting advertisements and online media (WeChat and Tencent QQ), with an average age of 20.12 ± 2.39 (M ± SD) years. All participants had normal/corrected to normal vision, and no history of brain trauma or mental illness. All of them were right-handed. None of them had participated in similar experiments before. This study was approved by the Research Ethics Committee of Yangzhou University (JKY-2022030508). All subjects signed the informed consent form before the experiment and received corresponding compensation after completing the experiment.

### 2.2. Materials

The experimental materials consisted of four-character words, facial images, and two-digit multiplication problems.

(1) Four-character words

Four hundred and fifty four-character words were divided into three categories according to their meanings, by 32 participants, i.e., view, reappraisal, and suppression words. In order to distinguish neutral facial expressions from neutral words, neutral words were named “view” (consistent with the “freely view” strategy in the field of emotion regulation). For a certain idiom, only if not less than 75% (24 participants) of the total number of participants classify it into one of the three groups mentioned above, the idiom can be used in the material of the formal experiment. Additionally, twenty-five college students were recruited to evaluate the familiarity of each idiom using a 9-point scale, where “1” means “very unfamiliar” and “9” means “very familiar”. Another twenty-five participants were asked to use a 9-point scale to evaluate the relevance of the meaning of each idiom to “describe natural scenes” (view), “none of my business” (reappraisal), and “hide one’s own emotional experience” (suppression), where “1” means “not relevant”, and “9” means “highly relevant”. Referring to previous studies [25,35], the fourth group of subjects (25 people) were asked to use a 9-point scale to evaluate their emotional experience elicited by each idiom. The two dimensions of valence and arousal are combined, where “1” means very negative, “5” means calm, “9” means very positive, from 5 to 1 means the negative emotional experience is getting stronger, from 5 to 9 means the positive emotional experience is getting stronger. All subjects who participated in the words assessment were no longer participating in the formal experiment. Finally, 72 words were selected as formal experimental materials, of which 40 were view related words, and 16 were reappraisal related words, and 16 were suppression related words.

Referring to previous study [27], the familiarity, relatedness to view, reappraisal, and suppression, as well as the emotional experience elicited by the three types of words, were analyzed. The results showed that (1) there was no significant difference between the participants’ familiarity towards the three types of words, F(2,69) = 1.401, *p* = 0.253. The familiarity towards the three groups of words was as follows: view, M ± SD, 7.70 ± 0.96; reappraisal, 8.06 ± 0.41; expression, 7.98 ± 0.65, indicating that the subjects were equally familiar with the three groups of words. (2) The relatedness between the words and “describe natural scenes”, “none of my business”, and “hide one’s own emotional experience”: A. There was a significant difference in the relatedness between the three types of words and “describe natural scenes”, F(2,69) = 2354.29, *p* < 0.001, the relatedness between the view words and “describe natural scenes” were significantly higher than (*ps* < 0.001) the reappraisal words and suppression words; there was no significant difference between the latter two (*p* > 0.05). B. There was a significant difference in the relatedness between the three types of words and “none of my business”, F(2,69) = 1083.09, *p* < 0.001, the relatedness between the reappraisal words and “none of my business” were significantly higher than (*ps* < 0.001) the view and suppression words, and the suppression words were higher than the view words (*p* < 0.001). C. There was a significant difference in the relatedness between the three types of words and “hide one’s own emotional experience”, F(2,69) = 1120.45, *p* < 0.001, the relatedness between the suppression words and “hide one’s own emotional experience” were significantly higher (*ps* < 0.001) than the view and reappraisal words, and the reappraisal words were higher than the view words (*p* < 0.001). The above results showed that there were significant differences among the three types of words in terms of the relatedness to any of the meanings “describe natural scenes”, “none of my business”, and “hide one’s own emotional experience”. (3) The relatedness between a certain idiom group and “describe natural scenes”, “none of my business”, and “hide one’s own emotional experience”: A. There was a significant difference between the relatedness of view words and “describe natural scenes”, “none of my business”, and “hide one’s own emotional experience”, F(2,45) = 4255.54, *p* < 0.001; the relatedness between view words and “describe natural scenes” were significantly higher (*ps* < 0.001) than the relatedness to “none of my business”, and “hide one’s own emotional experience”, and the difference between the relatedness of the latter two was not significant (*p* > 0.05). B. There was a significant differences between the relatedness of reappraisal words and “describe natural scenes”, “none of my business”, and “hide one’s own emotional experience”, F(2,45) = 962.27, *p* < 0.001; the relatedness between reappraisal words and “none of my business” were significantly higher (*ps* < 0.001) than with “describe natural scenes” and “hide one’s own emotional experience”, and the relatedness to “hide one’s own emotional experience” was higher than to “describe natural scenes” (*p* < 0.001). C. There was a significant difference between the relatedness between suppression words and “describe natural scenes”, “none of my business”, and “hide one’s own emotional experience”, F(2,45) = 561.38, *p* < 0.001; the relatedness between suppression words and “hide one’s own emotional experience” were significantly higher (*ps* < 0.001) than “describe natural scenes” and “none of my business”, and the difference between the relatedness to the latter two was not significant (*p* > 0.05). The above results showed that, for a specific group of words, there were significant differences between the relatedness to “describe natural scenes”, “none of my business”, and “hide one’s own emotional experience”. (4) There was no significant difference in the emotional experience elicited by the three types of words, F(2,69) = 0.89, *p* = 0.416, and the emotional experience intensity elicited by the three types of words were as follows: view, M ± SD, 5.16 ± 0.52; reappraisal, 5.15 ± 0.56; suppression, 5.35 ± 0.34.

(2) Facial images

The facial images used in the present study were selected from the NimStim Database [36]. Eighteen different facial identities were selected (9 women) (model numbers were as follows: woman: 1, 2, 3, 6, 9, 12, 13, 17, 19, man: 20, 21, 24, 27, 33, 36, 37, 41, 42) pictures, the images of each model expressed happy, neutral, and fear were adopted; a total of 54 facial images were used for the formal experiment. The valence and arousal of the three types of images were rated by 25 participants (did not participate in the formal experiment) using a 9-point rating scale. The results of repeated measures ANOVA (analysis of variance) showed that the main effect of the valence was significant, F(2,34) = 28.95, *p* < 0.001, η^2^ = 0.630. The valences of happy (M ± SD, 6.23 ± 1.12) images were significantly higher than those of fear (3.49 ± 0.84, *p* < 0.001) and neutral (4.61 ± 0.75, *p* = 0.005) images, and the valence of neutral images was significantly higher (*p* < 0.001) higher than for fear. The main effect of arousal was not significant, F(2,34) = 0.49, *p* = 0.617, η^2^ = 0.028. The arousals of happy, fearful, and neutral pictures were M ± SD, 5.62 ± 0.49, 5.70 ± 0.45 and 5.57 ± 0.50. All facial images were presented with the same visual angle (4.52° × 6.75°).

(3) Two-digit multiplication problems

A total of 54 two-digit multiplication estimation questions suitable for DU strategy were used. The principles for selecting the arithmetic problems were in accordance with previous studies [1,2,12]. The font of the estimation questions and alternative answer was Times New Roman, size 30 pt.

### 2.3. Procedure

**Figure 1:**
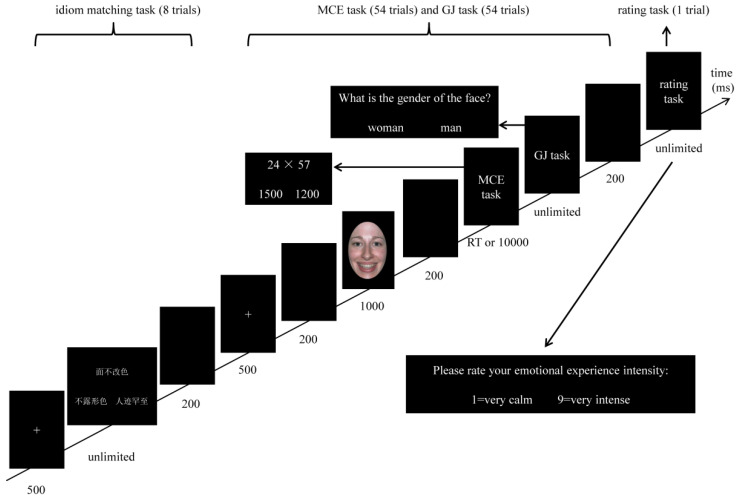
The illustration of single block. “面不改色” means keeping calm, “不露形色” means keeping calm, “人迹罕至” means desolate and remote places.

The experimental procedure was programmed with E-prime 2.0 (Psychology Software Tools Inc., Pittsburgh, PA, USA). The formal experiment consisted of 9 blocks, a single block sequentially containing the idiom matching task, MCE task, gender judgment task (GJ task), and emotional experience intensity assessment task (Figure 1). The specific details are as follows: (1) in the idiom matching task (8 times); a white”+” (500 ms); followed by three words (the target idiom at the top of the screen, while two alternative words were at the bottom of the screen, without time limit, until a response was given); then a blank (200 ms); participants were required to choose which alternative idiom conveyed the same meaning as the target idiom by pressing “F” (for the alternative idiom on the left side) or “J” (for the alternative idiom on the right side). (2) In the MCE task (54 times) and the GJ task (54 times), the order in which the stimuli were presented in a single trial was as follows: each trial started with a white “+” (500 ms), followed by a blank (200 ms), then the facial expression image (1000 ms), a blank (200 ms), the MCE task (RT or 10,000 ms), and next, the GJ task (unlimited), then a blank (200 ms). The facial expressions were administered in a randomized manner, and the probability of presenting each face expression image before the MCE and GJ task was equal. In the MCE task, participants were required to choose the correct answer by using the DU strategy, by pressing “F” (for the alternative answer on the left side) with their left middle finger, or pressing “J” (for the alternative answer on the right side) with their right middle finger. The alternative answers were calculated by DU and UD strategies. In the GJ task, participants were required to judge the gender of the facial expression by pressing “F” (for the alternative answer on the left side) with their left middle finger, or pressing “J” (for the alternative answer on the right side) with their right middle finger. Both in the MCE task and GJ task, the correct answer appears in different positions (on the left and right side) with the same probability. Participants were encouraged to complete both tasks as accurately and quickly as possible, though the GJ task had no time limit. (3) In the emotional experience intensity assessment task (at the end of each block), participants were required to rate their emotional experience intensity by using a 9-point scale: “1” means “very calm”, and “9” means “very strong”. Therefore, the whole experiment consisted of 72 times (9 blocks × 8 times/block) of the idiom matching task, 486 times (9 blocks × 54 trials/block) of MCE task, 486 times (9 blocks × 54 times/block) of GJ task, and 9 times of emotional experience intensity assessment (9 blocks × 1 time/block). Since the within-subject experimental design study was adopted in the present study, all participants were required to participate in the nine (3 implicit priming emotion type × 3implicit emotion regulation strategy type) different experimental conditions. Referring to the previous study [27], after completing the formal experimental tasks, participants were asked whether they realized the function of the idiom matching task. None of the participants found that the function of the idiom matching task was for implicit emotion regulation.

The present study was conducted in a quiet room. It was ensured that participants fully understood the experimental procedure; the eighteen practice trials idiom matching task, nine practice trials MCE task, nine practice trials GJ task, and one practice trials emotional experience intensity assessment task were provided before the formal test. Feedback was provided in the practice trials, while it was not provided in the formal experiment. The experimental materials (four-character words, facial images, and multiplication problems) used in the practice trials were not used in the formal experiment. To relieve fatigue, all participants were required to rest for 2 min after each block.

### 2.4. Data Recording and Analysis

The electrophysiological signals elicited by completing the task were recorded using Vision Recorder 2.0 (Brain Product, Gilching, Germany) with 64 scalp sites (10–20 system), while adapting FCz as the reference electrode. Both the horizontal and vertical electrooculogram (EOG) were recorded. The horizontal EOG was recorded from the electrode site at the outer canthi of the right eye, while the vertical EOG was recorded from the electrode placed 1 cm below the right eye. The electrode impedance was kept below 5 kΩ. The electrophysiological and EOG signals were amplified with a band pass of 0.01–100 Hz, and sampled at a rate of 500 Hz. Offline signal processing had been adopted to obtain a global average reference, by using Vision Analyzer 2.0 (Brain Product, Munich, Germany). The data were filtered with a 0.1 Hz high-pass cutoff and a 35 Hz low-pass cutoff (24 dB/octave), respectively. Trials with EOG artifacts (mean EOG voltage exceeding ±80 μV) were excluded from averaging. The EEG data were corrected for eye movements with ICA ocular correction. Noisy EEG sensors were identified through visual inspection, and the bad electrode was interpolated by using spline interpolation procedure. The TTL synchronization with stimuli presentation was adopted, which was based on a synchronization signal sent through a cable connection between the computer that was presenting the experimental materials, and the other computer that was recording the experimental data. Meanwhile, the TCP/IP Synchronization had been adopted. Using the TCP/IP connections between E-prime 2.0 and Vision Recorder 2.0 (Brain Product, Gilching, Germany) allowed us to send events from E-prime 2.0 to Vision Recorder 2.0. In the present study, different experimental conditions were defined by E-prime 2.0 before the experiment; thus, we could configure E-prime 2.0 to send an event at every onset of a stimulus. Additionally, event marking was adopted for post-processing synchronization.

The average epoch for ERP was 3000 ms, including a 200 ms baseline. According to previous studies [1,2,37], the onset was locked on the onset of the facial expression image. Trials were accepted for segmentation only if participants completed both the MCE and the GJ tasks correctly. EEG evoked by the angry, fear, neutral, and happy were averaged. In the present study, not only ERP components evoked by completing the MCE task (T-P1, T-N170, and T-LPC), but also ERP components evoked by processing the facial expression images (C-N170, and C-P3) were analyzed. Based on the topographical distribution of grand-averaged ERP activity of this study and previous studies [38,39], corresponding time windows and electrode sites for the ERP components related to the MCE task and facial expression processing were well extracted. To be more specific, (1) facial expression processing related components: the mean amplitude of C–N170 (140–180 ms) was analyzed at P5, P6, P7, P8, PO7, and PO8 electrode sites. (2) The MCE task-related components: the mean amplitude of T-P1 was analyzed at P5, P6, P7, P8, PO3, PO4, PO7, and PO8 electrode sites, during 90–120 ms after MCE task onset (corresponding to the time range of 1290–1320 ms post facial expression onset). The mean amplitude of T-N170 was analyzed at P5, P6, P7, P8, PO3, PO4, PO7, and PO8 electrode sites, during 140–180 ms after MCE task onset (corresponding to the time range of 1340–1380 ms post facial expression onset). The mean amplitude of T-LPC was analyzed at P7, P8, PO7, and PO8 electrode sites, during 600–1800 ms after MCE task onset (corresponding to the time range of 1800–3000 ms post facial expression onset). Referring to previous studies [13,20,26,40], in order to further examine the influence of dynamic process of emotion regulation on completing the MCE task, the LPC components were divided into three short time windows: LPC1 (1800–2200 ms), LPC2 (2200–2600 ms), and LPC3 (2600~3000 ms).

The ACC and RT of completing the idiom matching task and the MCE task, and the emotional experience intensity assessment score were analyzed by using ANOVA; the independent variables were implicit priming emotion type (happy, neutral, and fear) and type of emotion regulation strategies (neutral, reappraisal, and suppression). Additionally, three-factor repeated-measures ANOVA was performed on the ERP data, and the independent variables were implicit priming emotion type, type of emotion regulation strategies, and hemisphere (left hemisphere vs. right hemisphere). Dependent variables were the amplitudes of the ERP component mentioned above. SPSS 16.0 was used for statistical analysis, the Greenhouse–Geisser correction was applied to the *p* values, while Bonferroni’s correction was applied to post hoc tests. Additionally, partial eta-squared ($\eta_{p}^{2}$ ) was used to describe effect sizes.

## 3. Results

### 3.1. Behavioral Results

(1) The ACC and RT of completing the idiom matching task

ACC: The results of one-way ANOVA showed that the main effect of implicit emotion regulation strategy type was not significant, F(2,2087) = 1.631, *p* = 0.196. The ACC of completing the idiom matching task under view, implicit reappraisal, and implicit suppression conditions were as follows: M ± SD, 0.997 ± 0.054, 0.993 ± 0.085, and 0.999 ± 0.038, respectively. Additionally, the independent sample t-test results showed that the ACC of completing the idiom matching task under the above three conditions was significantly higher than (*ps* < 0.001) the random level (0.5).

RT: The results of one-way ANOVA showed that the main effect of implicit emotion regulation strategy type was significant, F(2,2043) = 33.812, *p* < 0.001. The RT of completing the idiom matching task under implicit reappraisal (M ± SD, 1423.609 ± 521.818) and implicit suppression (1389.248 ± 507.836) conditions were shorter (*ps* < 0.001) than under neutral (1624.857 ± 671.522) condition, while the RTs under the implicit reappraisal and implicit suppression conditions showed no significant difference (*p* > 0.05).

(2) The intensity of the emotional experience

The results of one-way ANOVA showed that the main effect of emotional experience intensity was significant, F(2,260) = 6.333, *p* = 0.002. Participants’ emotional experience intensity under view (M ± SD, 4.655 ± 1.904) conditions were higher than under implicit reappraisal (3.862 ± 1.825, *p* = 0.014) and implicit suppression (3.747 ± 1.767, *p* = 0.004) conditions, while their emotional experience intensity under the latter two conditions showed no significant difference (*p* > 0.05).

(3) Individual’s ACC and RT of completing the MCE task

**Figure 2:**
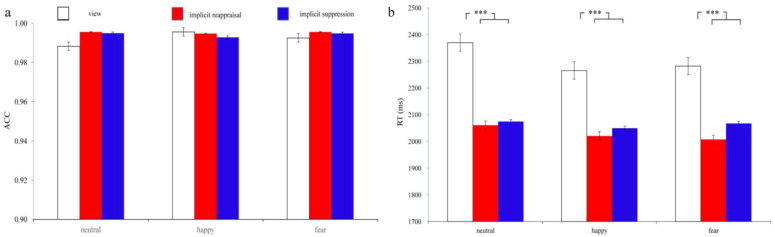
The ACC (**a**) and RT (**b**) of completing the MCE task under view (white bar), implicit reappraisal (red bar), and implicit suppression (blue bar) conditions. “***” means “*p* < 0.001”.

ACC: Two-way repeated-measure ANOVA showed that the main effect of implicit emotion regulation strategy type was not significant, F(2,50) = 1.727, *p* = 0.188, $\eta_{p}^{2}$ = 0.065. The main effect of implicit priming emotion type was not significant, F(2,50) = 0.358, *p* = 0.611, $\eta_{p}^{2}$ = 0.014. The interaction effect of these two factors was not significant, F(4,100) = 1.600, *p* = 0.203, $\eta_{p}^{2}$ = 0.060. The ACC under different conditions were shown in Figure 2a.

RT: Two-way repeated-measure ANOVA showed that the main effect of implicit emotion regulation strategy type was significant, F(2,50) = 16.427, *p* < 0.001, $\eta_{p}^{2}$ = 0.397. The post hoc test results showed that participants’ RT under view condition were longer (*ps* < 0.001) than under implicit reappraisal and implicit suppression conditions, while their RT under the latter two conditions showed no significant difference (*p* > 0.05). The main effect of implicit priming emotion type was not significant, F(2,50) = 3.016, *p* = 0.080, $\eta_{p}^{2}$ = 0.108. The interaction effect of these two factors was not significant, F(4,100) = 1.783, *p* = 0.138, $\eta_{p}^{2}$ = 0.067. The ACC under different conditions were shown in Figure 2b.

### 3.2. ERP Results

(1) ERPs elicited by processing facial expression

**Figure 3:**
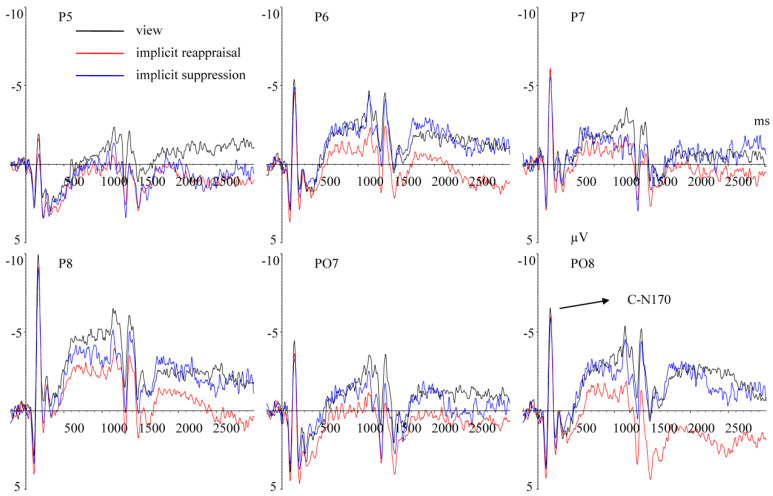
Grand average ERPs of C–N170 components elicited by completing the MCE task under view (black lines), implicit reappraisal (red lines), and implicit suppression (blue lines) conditions recorded at P5, P6, P7, P8, PO7, and PO8.

C–N170 (140–180 ms) Repeated measurement ANOVA revealed a significant main effect of hemisphere, F(1,25) = 12.719, *p* < 0.001, $\eta_{p}^{2}$ = 0.337. The C-N170 amplitudes in the right hemisphere (M ± SD, −5.653 ± 4.117 μV) were larger (*p* < 0.001) than in the left hemisphere (−2.871 ± 5.172 μV) (Figure 3). The main effect of implicit priming emotion type was not significant, F(2,50) = 1.044, *p* = 0.337, $\eta_{p}^{2}$ = 0.040. The main effect of implicit emotion regulation strategy type was not significant, F(2,50) = 1.393, *p* = 0.258, $\eta_{p}^{2}$ = 0.053. The interaction effect between implicit priming emotion type and implicit emotion regulation strategy type was not significant, F(4,100) = 1.051, *p* = 0.363, $\eta_{p}^{2}$ = 0.040. The interaction effect between implicit emotion regulation strategy type and hemisphere was not significant, F(2,50) = 0.057, *p* = 0. 944, $\eta_{p}^{2}$ = 0.002. The interaction effect between implicit priming emotion type and hemisphere was not significant, F(2,50) = 0.564, *p* = 0.573, $\eta_{p}^{2}$ = 0.022. The interaction effect between implicit priming emotion type, implicit emotion regulation strategy type, and hemisphere was not significant, F(4,100) = 1.681, *p* = 0.184, $\eta_{p}^{2}$ = 0.063.

(2) ERPs elicited by completing the MCE task

**Figure 4:**
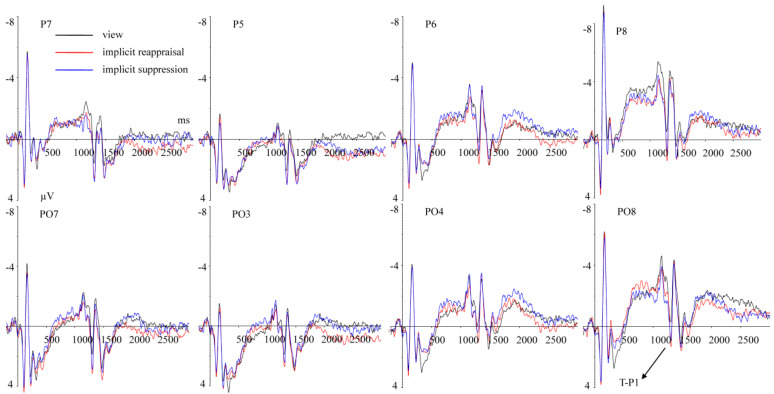
Grand average ERPs of T-P1 components elicited by completing the MCE task under view (black lines), implicit reappraisal (red lines), and implicit suppression (blue lines) conditions recorded at P5, P6, P7, P8, PO3, PO4, PO7, and PO8.

T-P1 (1290–1320 ms) Repeated measurement ANOVA revealed a significant main effect of implicit emotion regulation strategy type, F(2,50) = 9.758, *p* = 0.001, $\eta_{p}^{2}$ = 0.281. Compared with view (M ± SD, 0.503 ± 4.933 μV), the T-P1 amplitudes under implicit reappraisal (1.841 ± 3.356 μV, *p* = 0.001) and implicit suppression (1.483 ± 3.180 μV, *p* = 0. 044) conditions were larger (Figure 4). The main effect of hemisphere was significant, F(1,25) = 13.572, *p* = 0.001, $\eta_{p}^{2}$ = 0.352. The T-P1 amplitudes in the left hemisphere (2.293 ± 3.557 μV) were larger (*p* = 0.001) than in the right hemisphere (0.259 ± 4.089 μV) (Figure 4). The main effect of implicit priming emotion type was not significant, F(2,50) = 0.573, *p* = 0.499, $\eta_{p}^{2}$ = 0.022. The interaction effect between implicit priming emotion type and implicit emotion regulation strategy type was not significant, F(4,100) = 1.702, *p* = 0.199, $\eta_{p}^{2}$ = 0.064. The interaction effect between implicit emotion regulation strategy type and hemisphere was not significant, F(2,50) = 1.045, *p* = 0.359, $\eta_{p}^{2}$ = 0.040. The interaction effect between implicit priming emotion type and hemisphere was not significant, F(2,50) = 0.109, *p* = 0.897, $\eta_{p}^{2}$ = 0.004. The interaction effect between implicit priming emotion type, implicit emotion regulation strategy type, and hemisphere was not significant, F(4,100) = 2.555, *p* = 0.069, $\eta_{p}^{2}$ = 0.093.

**Figure 5:**
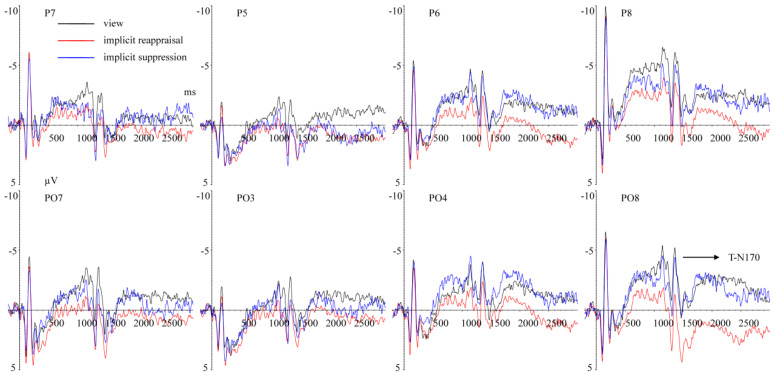
Grand average ERPs of T-N170 components for view (black lines), implicit reappraisal (red lines), and implicit suppression (blue lines), under implicit fear priming condition, recorded at P5, P6, P7, P8, PO3, PO4, PO7, and PO8.

**Figure 6:**
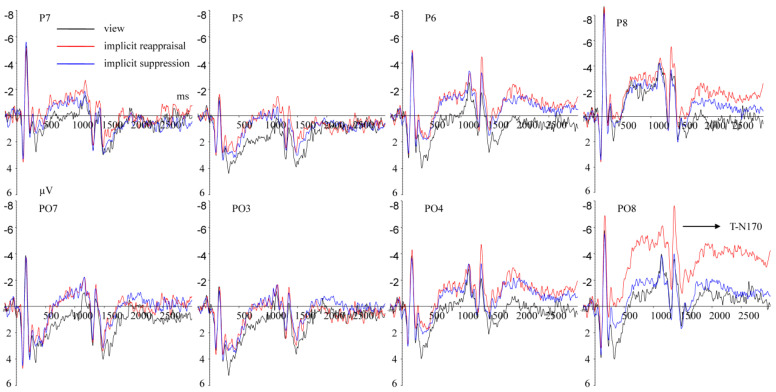
Grand average ERPs of T-N170 components for view (black lines), implicit reappraisal (red lines), and implicit suppression (blue lines), under implicit neutral priming condition, recorded at P5, P6, P7, P8, PO3, PO4, PO7, and PO8.

T-N170 (1340–1380 ms) Repeated measurement ANOVA revealed a significant main effect of hemisphere, F(1,25) = 19.004, *p* < 0.001, $\eta_{p}^{2}$ = 0.432; the T-N170 amplitudes in the left hemisphere (M ± SD, −1.597 ± 4.240 μV) were larger (*p* < 0.001) than in the right hemisphere (−3.568 ± 4.694 μV). The interaction effect between implicit priming emotion type and implicit emotion regulation strategy type was significant, F(4,100) = 3.234, *p* = 0.049, $\eta_{p}^{2}$ = 0.115. The results of the simple effects analysis show that A. under implicit fear priming condition, compared with implicit reappraisal (−1.056 ± 3.473 μV), the T-N170 amplitudes under view (−4.115 ± 6.121 μV, *p* = 0.027) and implicit suppression (−2.951 ± 3.183 μV, *p* = 0.014) conditions were larger (Figure 5); B. under implicit neutral priming condition, compared with view (−1.647 ± 6.000 μV, *p* = 0.047) and implicit suppression (−2.322 ± 3.148 μV, *p* = 0.036) conditions, the T-N170 amplitudes under implicit reappraisal (−3.988 ± 4.947 μV) condition were larger (Figure 6); C. under implicit happy priming condition, the T-N170 amplitudes related to different implicit emotion regulation strategy showed no significant difference (*ps* > 0.05). The main effect of implicit emotion regulation strategy type was not significant, F(2,50)= 0.828, *p* = 0.443, $\eta_{p}^{2}$ = 0.032. The main effect of implicit priming emotion type was not significant, F(2,50) = 0.122, *p* = 0.795, $\eta_{p}^{2}$ = 0.005. The interaction effect between implicit emotion regulation strategy type and hemisphere was not significant, F(2,50) = 1.386, *p* = 0.259, $\eta_{p}^{2}$ = 0.053. The interaction effect between implicit priming emotion type and hemisphere was not significant, F(2,50) = 0.132, *p* = 0.386, $\eta_{p}^{2}$ = 0.086. The interaction effect between implicit priming emotion type, implicit emotion regulation strategy type, and hemisphere was not significant, F(4,100) = 1.791, *p* = 0.191, $\eta_{p}^{2}$ = 0.067.

**Figure 7:**
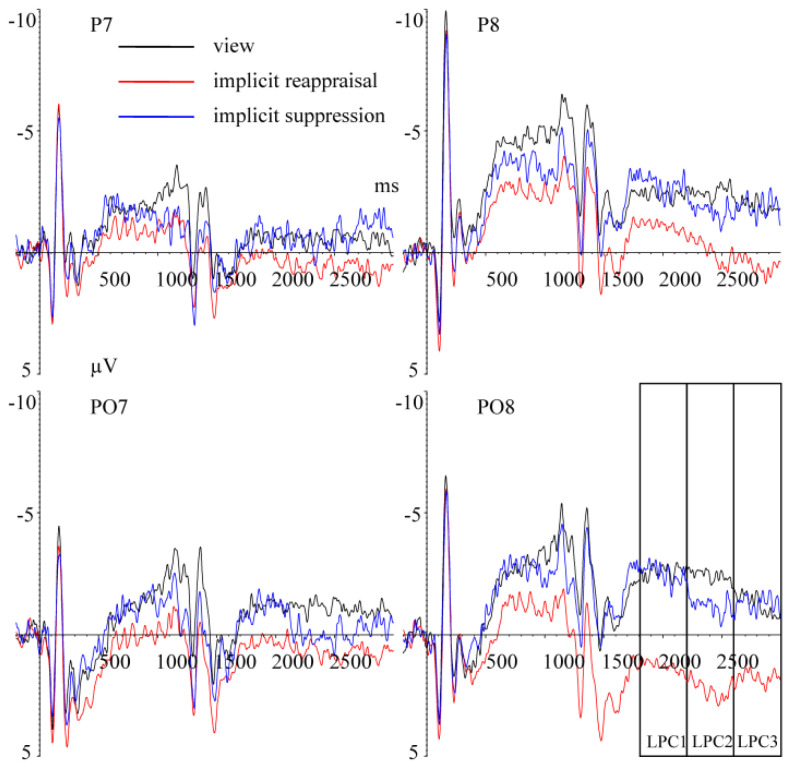
Grand average ERPs of T-LPC component for view (black lines), implicit reappraisal (red lines), and implicit suppression (blue lines), under implicit fear priming condition, recorded at P7, P8, PO7, and PO8.

T-LPC1 (1340–1380 ms) Repeated measurement ANOVA revealed a significant main effect of hemisphere, F(1,25) = 17.973, *p* < 0.001, $\eta_{p}^{2}$ = 0.418. The T-LPC1 amplitudes in the left hemisphere (M ± SD, −0.082 ± 3.584 μV) were larger (*p* < 0.001) than in the right hemisphere (−1.729 ± 4.818 μV). The main effect of implicit priming emotion type was not significant, F(2,50) = 0.172, *p* = 0.747, $\eta_{p}^{2}$ = 0.007. The main effect of implicit emotion regulation strategy type was not significant, F(2,50) = 1.083, *p* = 0.338, $\eta_{p}^{2}$ = 0.042. The interaction effect between implicit priming emotion type and implicit emotion regulation strategy type was not significant, F(4,100) = 2.185, *p* = 0.114, $\eta_{p}^{2}$ = 0.080. The interaction effect between implicit emotion regulation strategy type and hemisphere was not significant, F(2,50) = 0.318, *p* = 0.682, $\eta_{p}^{2}$ = 0.013. The interaction effect between implicit priming emotion type and hemisphere was not significant, F(2,50) = 0.318, *p* = 0.602, $\eta_{p}^{2}$ = 0.013. The interaction effect between implicit priming emotion type, implicit emotion regulation strategy type, and hemisphere was not significant, F(4,100) = 2.5911, *p* = 0.107, $\eta_{p}^{2}$ = 0.094 (Figure 7).

T-LPC2 (2200–2600 ms) Repeated measurement ANOVA revealed a significant main effect of hemisphere, F(4,100) = 12.808, *p* = 0.001, $\eta_{p}^{2}$ = 0.339; the T-LPC2 amplitudes in the left hemisphere (M ± SD, 0.240 ± 3.531 μV) were larger (*p* = 0.001) than in the right hemisphere (−1.171 ± 4.740 μV). The interaction effect between implicit priming emotion type and implicit emotion regulation strategy type was significant, F(4,100) = 3.261, *p* = 0.034, $\eta_{p}^{2}$ = 0.115. The results of the simple effects analysis show that A. under implicit fear priming condition, compared with implicit reappraisal (1.095 ± 3.915 μV), the T-LPC2 amplitudes under view (−1.871 ± 4.565 μV, *p* = 0.016) and implicit suppression (−0.957 ± 3.310 μV, *p* = 0.028) conditions were smaller (Figure 7); B. under implicit neutral and happy priming condition, the T-LPC2 amplitudes related to different implicit emotion regulation strategy showed no significant difference (*ps* > 0.05). The main effect of implicit emotion regulation strategy type was not significant, F(2,50) = 1.812, *p* = 0.182, $\eta_{p}^{2}$ = 0.068. The main effect of implicit priming emotion type was not significant, F(2,50) = 0.198, *p* = 0.708, $\eta_{p}^{2}$ = 0.008. The interaction effect between implicit emotion regulation strategy type and hemisphere was not significant, F(2,50) = 0.380, *p* = 0.635, $\eta_{p}^{2}$ = 0.015. The interaction effect between implicit priming emotion type and hemisphere was not significant, F(2,50) = 0.705, *p* = 0.427, $\eta_{p}^{2}$ = 0.027. The interaction effect between implicit priming emotion type, implicit emotion regulation strategy type, and hemisphere was not significant, F(4,100) = 2.205, *p* = 0.138, $\eta_{p}^{2}$ = 0.081.

T-LPC3 (2600–3000 ms) Repeated measurement ANOVA revealed a significant main effect of hemisphere, F(4,100) = 7.288, *p* = 0.012, $\eta_{p}^{2}$ = 0.226; the T-LPC3 amplitudes in the left hemisphere (M ± SD, 0.191 ± 4.013 μV) were larger (*p* = 0.001) than in the right hemisphere (−0.958 ± 4.529 μV). The interaction effect between implicit priming emotion type and implicit emotion regulation strategy type was significant, F(4,100) = 3.127, *p* = 0.032, $\eta_{p}^{2}$ = 0.111. The results of the simple effects analysis show that A. under implicit fear priming condition, compared with implicit reappraisal (1.146 ± 3.282 μV), the T-LPC3 amplitudes under view (−1.394 ± 4.372 μV, *p* = 0.014) and implicit suppression (−1.268 ± 4.225 μV, *p* = 0.009) conditions were smaller (Figure 7); B. under implicit neutral and happy priming condition, the T-LPC3 amplitudes related to different implicit emotion regulation strategy showed no significant difference (*ps* > 0.05). The main effect of implicit emotion regulation strategy type was not significant, F(2,50) = 0.655, *p* = 0.524, $\eta_{p}^{2}$ = 0.026. The main effect of implicit priming emotion type was not significant, F(2,50) = 0.709, *p* = 0.452, $\eta_{p}^{2}$ = 0.028. The interaction effect between implicit emotion regulation strategy type and hemisphere was not significant, F(2,50) = 0.312, *p* = 0.734, $\eta_{p}^{2}$ = 0.012. The interaction effect between implicit priming emotion type and hemisphere was not significant, F(2,50) = 1.302, *p* = 0.271, $\eta_{p}^{2}$ = 0.050. The interaction effect between implicit priming emotion type, implicit emotion regulation strategy type, and hemisphere was not significant, F(4,100) = 2.202, *p* = 0.131, $\eta_{p}^{2}$ = 0.081.

In order to enhance readability, the comparisons of ERP amplitudes induced by different experimental conditions were summarized (see Table 1 for details).

## 4. Discussion

Adopting the ERP technique, combined with the implicit emotion priming paradigm (GJ task), participants were asked to complete the idiom matching task (an implicit emotion regulation method). Meanwhile, they were asked to complete the MCE task with the DU strategy. The present study explored the relationship between implicit emotion regulation and an individual’s estimation performance. The behavioral results showed that participants completed the idiom matching task carefully, and that implicit reappraisal and implicit suppression equally reduced an individual’s emotional experience intensity. Both implicit reappraisal and implicit suppression contributed to reducing an individual’s RT (but not ACC) of completing the MCE task. In addition, the ERP results showed that both the encoding of the estimation question (T-P1 and T-N170) and the retrieval of arithmetic facts (T-LPC) were influenced by implicit emotion regulation.

### 4.1. Implicit Emotion Regulation Manipulation and Emotional Experience Intensity

A large number of previous studies have demonstrated that the idiom matching task was an effective way of implicit emotion regulation [25,26,41,42]. The experimental results showed that participants’ ACCs of completing the idiom matching task under all conditions were higher than 0.99, which were significantly higher than the probability level (0.5), and there was no significant difference between any two conditions, indicating that the participants completed the idiom matching task seriously.

The emotional experience intensity score-related results showed that, compared with the baseline level (view condition), implicit reappraisal and implicit suppression effectively reduced participants’ emotional experience intensity after completing the MCE task, and the regulation effect of implicit reappraisal and implicit suppression were equivalent. A previous study showed that through implicit emotion regulation, an individual’s anger experience can be reduced [23], and the other studies [25,27,43] indicated that implicit emotion regulation can effectively reduce a participant’s emotional experience intensity when they complete the decision task and facial expression identify task, which supported our results to some extent. Therefore, both previous studies and the present study consistently indicated that implicit emotion regulation can effectively reduce an individual’s emotional experience intensity when they were required to complete different cognitive tasks, which supported the dual-process framework theory [21].

### 4.2. The Influence of Implicit Emotion Regulation on the ACC and RT of Completing the MCE Task

Consistent with previous study [12], the present study showed that participants’ ACCs of completing the MCE task under different implicit emotion regulation conditions showed no significant difference, which indicated that their ACCs of completing the MCE task were not influenced by implicit emotion regulation. A recent study [31] showed that high (vs. low) math anxious individuals showed decreases in math accuracy associated with increased electrodermal activity under suppression conditions. However, for both high and low math anxious individuals, reappraisal weakened the relationship between physiological arousal and math accuracy, and even elevated physiological arousal was no longer negatively related with math accuracy, which suggested that reappraisal (but not suppression) contributed to improving a math anxious individual’s math accuracy, which supported our findings to some extent. Considering explicit emotion regulation had been adopted in Pizzie and Kraemer’s work [31], and participants’ math anxiety levels had been measured, we proposed that the regulation effect of suppression on math accuracy would be affected by math anxiety level and emotion regulation style (explicit vs. implicit), while the regulation effect of reappraisal was relatively stable.

Additionally, the present study found that a participant’s reaction speed of completing the MCE task was quicker under implicit reappraisal and implicit expression conditions, than under view conditions, and their reaction speed under implicit reappraisal and implicit expression conditions showed no significant difference; these results seemed to indicate that implicit emotion regulation could provide more efficient processing for arithmetic tasks, through modulating the cognitive and emotional control capacities, thus leading to a faster response speed under implicit reappraisal and implicit suppression conditions which was consistent with a previous study [44]. Totally speaking, the present study showed that RT (vs. ACC) was a sensitive indicator to examine the influence of implicit emotion regulation on an individual’s estimation performance. Hypothesis one was confirmed.

### 4.3. The Potential Mechanisms of Implicit Facial Expression Processing

A large number of previous studies [45,46,47] indicated that the GJ task could successfully induce implicit emotional experience when facial expression images were used as emotional stimuli. Therefore, the GJ task has been adopted in this study to achieve the purpose of implicit emotion priming. In fact, after the formal experiment ended, we also verbally asked all participants how they thought about the purpose of setting the GJ task; none of them reported that the task was adopted to induce their implicit emotional experience, which indicated that we successfully achieved the purpose of implicit emotion priming.

The present study showed that the C-N170 amplitudes elicited by processing different facial expression images (happy, neutral, and fear) showed no significant difference. Previous studies showed that N170 was an effective indicator of facial expression processing [39,48], and the N170 amplitudes elicited by emotional (vs. neutral) facial expressions were larger [47,49,50], which was inconsistent with our results. This inconsistency may be caused by different experimental tasks, i.e., explicit facial expression processing tasks were adapted in studies mentioned above, while the implicit facial expression processing task was adopted in the present study. However, when completing implicit facial expression processing tasks, the N170 amplitudes elicited by emotional and neutral facial images showed no significant differences [16,46], which were consistent with our results. Additionally, the results of present study showed that compared with the left hemisphere, the C-N170 amplitudes in the right hemisphere were larger, which was supported by previous studies [47,51,52].

### 4.4. The Potential Brain Mechanisms of Estimation Performance Influenced by Implicit Emotion Regulation

The present study showed that when participants complete the MCE task with the DU strategy under different implicit emotion regulation conditions, the T-P1, T-N170, and T-LPC amplitudes are elicited, indicating that the different stages of individuals completing the MCE task are affected by implicit emotion regulation. The present study showed that the T-P1 amplitudes elicited by completing the MCE task were larger in the left (vs. right) hemisphere, suggesting that the left hemisphere was more sensitive to completing digit processing tasks, which was consistent with previous studies [53,54]. In terms of the types of implicit emotion regulation strategy types, compared with the baseline (view), the T-P1 amplitudes elicited by completing the MCE task under implicit reappraisal and implicit suppression condition were larger, suggesting that implicit emotion regulation enhanced an individual’s encoding sensitivity to the estimation question. This can be related to previous studies indicating that the P1 component was an effective indicator of indexing attention to digit-pattern, and that their potential numeric meaning increased attention resources along larger P1 amplitudes [1,15,16].

The present study showed that the T-N170 amplitudes were influenced by the interaction effect of implicit priming emotion type and implicit emotion regulation strategy type. To be more specific, compared with the baseline (view) and implicit suppression, implicit reappraisal contributed to estimation question encoding (smaller T-N170 amplitudes) under the implicit neutral priming condition. However, inverse results were found under the implicit fear priming condition, while an individual’s encoding performances were not influenced by implicit emotion regulation under the implicit happy priming condition. Totally speaking, both T-P1 and T-N170 belong to the ERP components induced in the encoding stage of the MCE task; T-N170 (but not T-P1) amplitudes were influenced by the interaction effect of implicit priming emotion type and implicit emotion regulation strategy type, which clearly reveal that the dynamic process of encoding are modulated by implicit emotion regulation. These results indicate the superiority of analyzing several small time windows (T-P1 and T-N170) separately at the encoding stage, instead of adapting a general analysis of the ERP amplitude changes over a longer period of time window, such as 0–200 ms [55,56], 0–250 ms, and so on [57].

LPC is a positive component that appears in the visual cortex during the late stages of processing the target stimuli, and it is often regarded as an effective indicator of resource allocation in response to attention [58,59,60]. The present study showed that implicit emotion regulation dynamically affected attention allocation while individuals were completing the MCE task. In the early stage of estimation operation (T-LPC1), a participant’s estimation performance was not influenced by implicit emotion regulation, reflected by the T-LPC1 amplitudes showing no significant differences under different implicit emotion regulation conditions. Meanwhile, in the LPC2 and T-LPC3 stages, the influence of implicit emotion regulation on an individual’s estimation performance was consistent. In detail, the T-LPC2 and T-LPC3 amplitudes were larger when individuals were using the implicit reappraisal (vs. view, implicit suppression) under the implicit fear priming condition. In addition, the corresponding T-LPC2 and T-LPC3 amplitudes under view and implicit suppression conditions showed no significant differences, indicating greater attention resources cost under implicit reappraisal (but not implicit suppression) condition, reflecting the superiority effect of implicit suppression (vs. implicit reappraisal). It should be noted that this superiority effect only occurred under the implicit fear priming condition, which also indicated that the influence of implicit emotion regulation on an individual’s estimation performance was modulated by the valence of the implicit priming stimulus. Additionally, the present study showed that both the T-LPC2 and T-LPC3 amplitudes were influenced by implicit emotion regulation, while the T-LPC1 amplitudes were not, suggesting the necessity of dividing LPC into three distinct time windows (LPC1, LPC2, and LPC3), and this was consistent with previous studies [13,20,26,28,40]. Hypothesis two was partly confirmed.

To sum up, the behavioral results of this study showed that participants successfully carried out implicit emotion regulation by completing the idiom matching task, and participant’s RT (but not ACC) of completing the MCE task with the DU strategy was influenced by implicit emotion regulation. Implicit reappraisal and implicit suppression equally contributed to improving a participant’s estimation performance under different implicit priming conditions. The ERP results revealed the dynamic time course of implicit emotion regulation affecting estimation performance. Firstly, in the encoding stage, the influence of implicit reappraisal on encoding sensitivity was increased first (larger T-P1 amplitudes) and then decreased (small T-N170 amplitudes), while implicit suppression contributed to improving a participant’s encoding sensitivity (larger T-P1 amplitudes). Secondly, in the retrieval stage, implicit reappraisal (vs. view) cost more attention resources (larger T-LPC2 and T-LPC3 amplitudes) when completing the MCE task under the implicit fear priming condition, which reflected the advantage effect of implicit expression (vs. implicit reappraisal).

The present study investigated the influence of implicit emotion regulation on an individual’s estimation performance under implicit emotional priming condition for the first time. The behavioral results showed that an individual’s estimation performances were equally improved (shorter RTs) by implicit reappraisal and implicit suppression, while the ERP results further expanded our understanding of the relationship between implicit emotion regulation and an individual’s estimation performance. In detail, both implicit suppression and implicit reappraisal contributed to improving an individual’s estimation performance, and the regulation effect of implicit suppression was better than implicit reappraisal, especially under the implicit fear priming condition. However, it should be noted that the ACCs around 0.99 suggested that the difficulty of the estimation task may have been too low in order to allow variations, generated by the emotion regulation strategy, to be induced. The behavioral results showed that an individual’s ACCs of completing the MCE task were not influenced by the implicit emotion prime and implicit emotion regulation; this may be related to the fact that only one estimation strategy has been adopted in the present study, and future studies can verify this speculation by adopting harder estimation strategies (e.g., RD and RU).

## 5. Conclusions

The present study revealed that an individual’s estimation performance was modulated by implicit emotion regulation. Compared with view, both implicit reappraisal and implicit suppression contributed to reducing an individual’s RT of completing the MCE task. Implicit reappraisal and suppression were equivalent in reducing an individual’s emotional experience intensity when they completed the MCE task. Compared with view, implicit reappraisal first enhanced (larger T-P1 amplitudes) and then weakened (smaller T-N170 amplitudes) an individual’s encoding sensitivity, while implicit suppression only enhanced an individual’s encoding sensitivity (larger T-P1 amplitudes). Additionally, under implicit fear priming condition, compared with view, there was implicit reappraisal (but not implicit suppression) along with more attention resource cost (larger T-LPC2 and T-LPC3 amplitudes). Totally speaking, both implicit reappraisal and implicit suppression contributed to improving an individual’s estimation performance, and the regulation effect of implicit suppression (vs. implicit reappraisal) was better.

## Figures and Tables

**Table 1:** The comparisons of ERP amplitudes of completing the MCE task under different experimental conditions.

	Neutral	Happy	Fear
T-P1	r,s > v	r,s > v	r,s > v
T-N170	r > v,s	ns	r < v,s
T-LPC1	ns	ns	ns
T-LPC2	ns	ns	r > v,s
T-LPC3	ns	ns	r > v,s

Note: “>” means larger ERP amplitudes. “v” = ”view”, “r” = ”implicit reappraisal”, “s” = “implicit suppression”, “ns” = ”not significant”. For example, under neutral priming condition, the T-P1 related results showed that “r,s > v”, suggesting under neutral priming condition, compared with view, the T-P1 amplitudes elicited by completing the MCE task under implicit reappraisal and implicit suppression condition were larger.

## Data Availability

The data used to support the findings of this study are available from the corresponding author upon request. The data are not publicly available due to privacy or ethical restrictions.
